# Effect of a customized digital adherence tool on retention in care and adherence to antiretroviral treatment in breastfeeding women, children and adolescents living with HIV in Tanzania: a mixed-methods study followed by clinical trials

**DOI:** 10.1186/s13063-023-07293-1

**Published:** 2023-04-21

**Authors:** I. Marion Sumari-de Boer, Kennedy M. Ngowi, Iraseni U. Swai, Lyidia V. Masika, Rehema A. Maro, Alan E. Mtenga, Benson A. Mtesha, Pythia T. Nieuwkerk, Ria Reis, Tobias F. Rinke de Wit, Rob E. Aarnoutse

**Affiliations:** 1 Kilimanjaro Clinical Research Institute (KCRI), PO Box 2236, Moshi, Tanzania; 2 Amsterdam Institute for Global Health and Development, Amsterdam, the Netherlands; 3 Institute of Public Health, Kilimanjaro Christian Medical University College, Moshi, Tanzania; 4 Knowledge, Technology & Innovation Group, Wageningen University & Research, Wageningen, the Netherlands; 5 Department of Medial Psychology, Amsterdam UMC, Amsterdam, the Netherlands; 6 Department of Global Health, Amsterdam UMC, Amsterdam, the Netherlands; 7 The Children’s Institute, University of Cape Town, Cape Town, South Africa; 8 Department of Pharmacy, Radboud UMC, Nijmegen, the Netherlands

**Keywords:** HIV, Adherence, Digital Health, Children, Adolescents, Breastfeeding women

## Abstract

**Background:**

Adherence to antiretroviral (ARV) treatment for HIV infection is challenging because of many factors. The World Health Organization (WHO) has recommended using digital adherence technologies (DATs). However, there is limited evidence on how DATs improve adherence. Wisepill® is an internet-enabled medication dispenser found feasible and acceptable in several studies. However, limited evidence is available on its effectiveness in improving ART adherence, specifically among children and adolescents. Furthermore, DATs are often developed without involving the target groups. We propose a two-stage project consisting of a formative study to customize an existing Wisepill DAT intervention and a randomized clinical trial to investigate the effectiveness of DAT combined with reminder cues and tailored feedback on adherence to ARV treatment among children and adolescents living with HIV and retention in care among breastfeeding women living with HIV in Kilimanjaro and Arusha Region, Tanzania.

**Methods:**

We will conduct a formative mixed-methods study and three sub-trials in Kilimanjaro and Arusha Regions among (1) children aged 0–14 years and their caregivers, (2) adolescents aged 15–19 years and (3) breastfeeding women and their HIV-negative infants. In the formative study, we will collect and analyse data on needs and contents for DATs, including the contents of short message service (SMS) texts and tailored feedback. The results will inform the customization of the DAT to be tested in the sub-trials. In the trials, participants will be randomized in the intervention arm, where the DAT will be implemented or the control arm, where standard care will be followed. Participants in the intervention arm will take their medication from the Wisepill box and receive daily reminder texts and tailored feedback during clinic visits.

**Discussion:**

If the intervention improves adherence to ART and the devices are acceptable, accurate and sustainable, the intervention can be scaled up within the National Aids Control Programmes.

**Trial registration:**

PACTR202301844164954, date 27 January 2023.

## Introduction

Children and adolescents form a significant part of the world population of people living with human immunodeficiency virus (HIV), with the World Health Organization (WHO) reporting 1.8 million children (age < 15) and 1.8 million adolescents (ages 10–19) in Sub-Saharan Africa in 2019 [1]. In addition, new infections continue to occur. In 2019, an estimated 160,000 children and 160,000 adolescents got infected globally [1]. The ‘START FREE-STAY FREE-AIDS FREE’ ‘superfast’ framework developed by The Joint United Nations Programme on HIV/AIDS (UNAIDS) has set new targets for an acquired immunodeficiency syndrome (AIDS)-free generation focusing on those aged 0–24 [2]. These targets highly depend on adherence to treatment and retention in care of pregnant and breastfeeding women, children and adolescents living with HIV. In 2017 Sub Saharan Africa (SSA), 81% of pregnant and breastfeeding women had access to treatment, with only 49% access for children aged 0–14 [3]. A limited number of people in SSA is virologically suppressed, with an overall percentage of 53% in 2018 [4]. This low percentage is mainly caused by limited treatment adherence and care retention [3]. High levels of adherence, i.e., taking more than 95% of prescribed doses, are needed to prevent treatment failure (defined as viral load becoming detectable, development of opportunistic infections or mortality), the development of HIV drug resistance and to prevent vertical transmission of HIV to the newborn child [5, 6]. Retention into care is a prerequisite for adherence to treatment. Reasons for non-retention are stigma, fear of disclosure and lack of social support [7].

Despite high access for pregnant women to HIV treatment of 92%, the percentage of infected infants has not decreased much since 2010 [8]. In Tanzania, 11% of infants born to women living with HIV are HIV-positive. Five percent of infections in children happen during the first 6 weeks of life [8]. Although the National Aids Control Programme (NACP) of Tanzania encourages monthly antenatal care (ANC) visits for pregnant women living with HIV [9], a study in Kilimanjaro, Tanzania, showed that retention in care is limited, with 59% lost to follow-up, of whom 19% in the first month after HIV diagnosis, 12% during pregnancy, and 70% after delivery [10].

It is estimated that only 59% of children (0–14 years) living with HIV are on anti-retroviral (ARV) treatment in Eastern and Southern Africa [3]. In Tanzania, 110,000 children lived with HIV in 2019, with 56% on treatment [1]. Children living with HIV must take lifelong treatment; most depend on their parents/caretakers regarding adherence to treatment. A study in East Africa showed that parents of 90% of children reported good adherence, though this study only used self-report and pill counts. Therefore, it was recommended that consistent adherence monitoring with validated measures and attention to vulnerable groups is needed [11]. A study in Dar es Salaam among orphaned children aged 2–14 showed that 72% reached 95% adherence based on nevirapine plasma levels. Challenges for adherence were financial constraints, waiting times at clinics and limited knowledge about HIV among caretakers [12]. Another study in Mwanza, Tanzania, among children and adolescents also reported limited adherence of 65% based on pill counts [13].

Adolescents (aged 15–19) are more independent than children below 14 and depend less on their parents. Reports from nurse counsellors in our setting imply that older children, including adolescents, visit clinics independently and often do not have their parents/caregivers as treatment supporters. Virological suppression is more difficult in this group, and levels differ significantly between studies: 27 to 89% [14]. Adherence among adolescents shows a variety between 64 and 90% [15]. Factors associated with poor adolescent adherence are stigma, adverse effects to antiretroviral treatment, lack of assistance and forgetfulness [15]. In Kilimanjaro, a study reported that 40% of adolescents had a virological failure, and about one-third reported poor adherence [16]. A study in Mwanza showed roughly the same, with only 65% of children and adolescents reaching adequate levels of adherence [13]. No single intervention has been proven effective among adolescents in low- to middle-income countries [17]. It was shown that phone-based interventions might be promising, but more rigorous studies are needed [18].

Digital adherence tools (DATs) have been proposed for monitoring adherence and have been investigated in several studies [19–32]. In the Kilimanjaro region, we have investigated the use of real-time medication monitoring by using the Wisepill® device/pillbox [33]. Wisepill® records the opening of the pillbox, a so-called medication event, and sends a short message service (SMS) text to patients if the pillbox is not opened on time to remind them. We showed that using the pillbox is feasible and that adult HIV-infected patients saw it as a good means for helping them adhere to and store their medication [19]. Numerous studies have shown evidence of using SMS among pregnant women living with HIV to improve retention into care and adherence to treatment [20, 25–30]. Our pilot study among pregnant and breastfeeding women living with HIV has shown feasibility and confirms positive user experience with SMS reminder cues [31, 32]. In another study, we found that the percentage of participants reaching 90% adherence was significantly higher for those using DATs [21]. We also encountered concerns about unwanted disclosure and stigma due to SMS’s explicit contents [22, 23]. In addition, there have been technical challenges, such as limited network availability [24].

Using the common way of communication through SMS has a high potential for disease management. The number of mobile phone subscriptions per 100 inhabitants in Tanzania was 86% in 2020. Additionally, over 25 billion local SMS were sent in the second quarter of 2019 [34]. In our previous studies, we found that using real time medication monitoring (RTMM) and SMS was feasible for reminding about medication intake among adults in Moshi, Tanzania [19, 31, 32]. In addition, studies in Kenya have shown SMS increased communication between patients and health care providers and retention in care and adherence to treatment [35–37]. Despite positive results, several studies have reported no clear effect on clinical outcomes [38]. In rural Malawi, an SMS trial could not show an effect of SMS on retention to care for people living with HIV [39]. Studies have suggested interventions to be developed through formative studies whereby the target population is engaged in designing the interventions early in the project [40–42].

Only limited evidence exists for interventions improving adherence to treatment and retention in care for children and adolescents with HIV in SSA [3]. As the number of mobile phone users is high and still increasing, particularly in Tanzania, this creates a better avenue for digital health tools to be integrated with HIV care for improving adherence to treatment and retention into care. Therefore, in this study, we propose to investigate using a customized DAT to improve retention in care, adherence to treatment and subsequent treatment outcomes among infants of breastfeeding mothers living with HIV and children and adolescents living with HIV.

## Methods/design

### Study area

We will conduct our project in care and treatment clinics (CTC) and postnatal clinics serving clients on antiretroviral treatment in the Kilimanjaro region in Tanzania, which has an area of 13,250 km^2^. The region has seven districts, and its capital is Moshi. In the latest census of 2012, the region had a population of about 1.6 million. Seventy-six percent live in rural areas, and 38% are under 15.

### Design

**Fig. 1:**
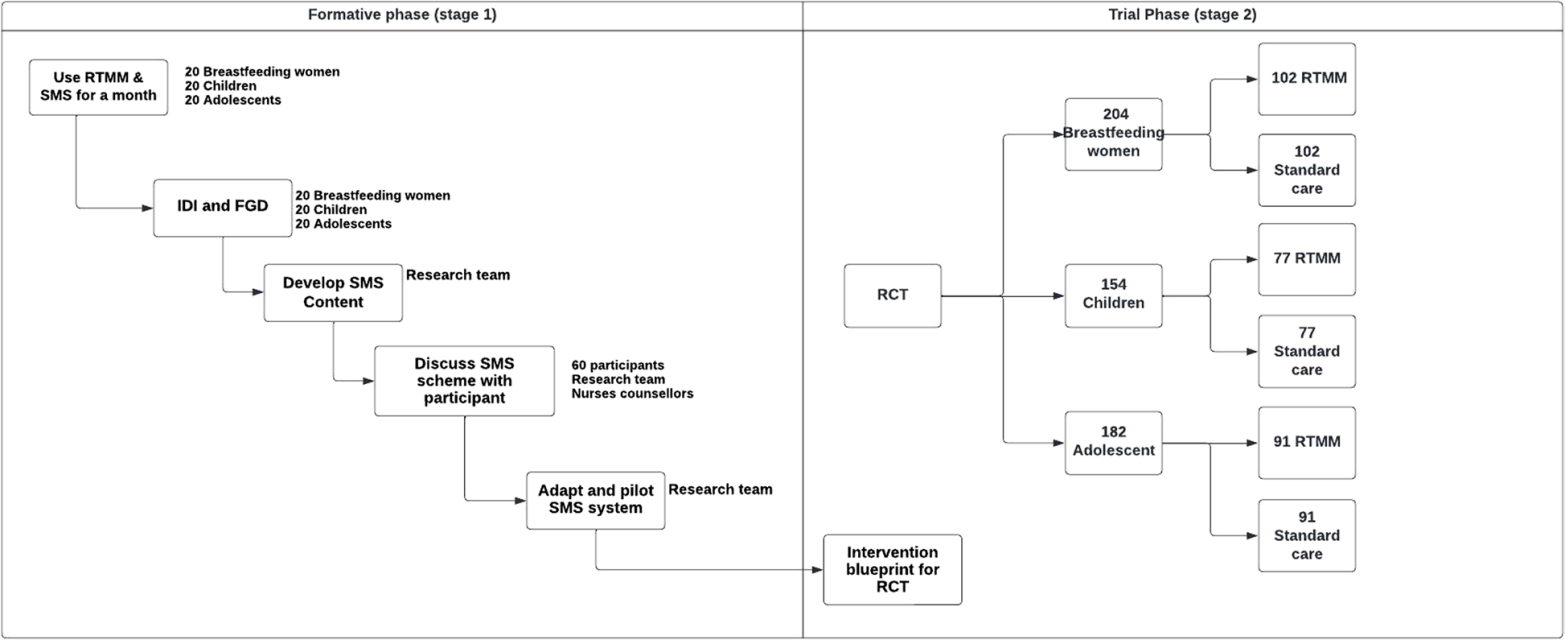
Schematic display of the study

This project will evaluate a DAT consisting of real-time medication monitoring with the Wisepill® device and SMS messages and tailored feedback to participants on their adherence data. The project will consist of two stages (see Fig. 1). One is a formative stage in which we will determine the contents and timing of the DAT. This will consist of a mixed methods approach. After using our DAT, we will conduct semi-structured interviews and qualitative data collection, including in-depth interviews and focus group discussions. The results of these data will inform the contents of the DAT to be used for each group. In the second stage, we will conduct three clinical trials to investigate the effect of the digital health intervention on adherence to treatment, retention into care and treatment outcomes among (1) breastfeeding women, (2) children and (3) adolescents.

### Overall aim and objectives

We aim to investigate whether our customized digital adherence tool will improve retention in care among breastfeeding women and adherence to treatment among children and adolescents.

The specific objectives for the first stage are as follows:

1. To identify the enablers and barriers to using DAT among children and their caregivers/parents, adolescents and breastfeeding women living with HIV in Kilimanjaro, Tanzania
2. To identify the needed content of SMS schemes based on the participants’ preferences
3. To identify the preferred timing and frequency for SMS, including reminder cues to take medication and to visit the clinic
4. To understand the need for additional information for different groups to be included in the SMS, i.e. education on sexual and reproductive health importance of breastfeeding, amongst others
5. To design the blueprint for the DAT to be tested in the second phase of the project

In the second stage of clinical trials, we will investigate the effect of our developed DAT according to the blueprint. This will be established through the following specific objectives:

1. Breastfeeding women: to assess the percentage retention in care at 18 months post-partum
2. Children: to assess the percentage of children being 95% adherent to treatment based on pharmacy refill counts and self-reported adherence
3. Adolescents: to assess the percentage of adolescents being 95% adherent to treatment based on pharmacy refill counts and self-reported adherence

Our secondary objectives for the second stage are as follows:

1. To assess the percentage of breastfeeding women, children and adolescents living with HIV reporting 95% adherence at 18, 12 and 12 months, respectively
2. To assess the percentage of breastfeeding women, children and adolescents living with HIV being virologically suppressed (<20 copies/ml) at 18, 12 and 12 months, respectively
3. To determine the trend in adherence over time based on self-report and pharmacy refill counts for breastfeeding women, children and adolescents living with HIV
4. To establish different cut-off points of adherence levels and their association with virological suppression
5. To determine the HIV status of the infants of breastfeeding women living with HIV at 6 weeks, 9 months and 18 months
6. To investigate the feasibility and acceptability of the DAT
7. To determine the implementation costs of the DAT

### Formative stage

The objective of this stage is to evoke feedback from the key intervention stakeholders to guide on designing and developing the newly proposed DAT. We will first survey 142 participants from each group to investigate enablers and barriers to using DAT. This figure is based on selecting an arbitrary percentage of 50% experiencing barriers for using DAT with *α* = 0.05 and using a power of 80% for estimating a single proportion sample calculation.

Next, we will purposively select 20 participants from each group, considered enough to reach ‘saturation’ of information, to use the DAT for 1 month. After signing informed consent, we will provide mothers, parents/caregivers of children and adolescents with a Wisepill® box which they will use for a month. After a month, we will interview each of them, including the children whose status has been disclosed on their experience with the box and the need for SMS content to be developed for the customized digital tool. We will also hold two focus group discussions with each group of breastfeeding mothers, parents/caregivers and adolescents. The outcomes of the formative research will be as follows:

1. An overview of needs for SMS contents and timing for reminder cues to take medication
2. An overview of needs for SMS contents and timing for reminder cues for clinic visits
3. An overview of further needs for other SMSs, such as educational and informational texts, including timing and frequency
4. An overview of enablers and barriers to the success of the intervention

Quantitative data from the survey will be analysed descriptively using frequency tables, chi-square tests and *t*-tests to investigate differences between subgroups. We will do thematic content analyses of qualitative data to understand the context of adherence to treatment and the use of DAT in the different target groups. We will use NVivo® software for data organization and define themes and subthemes. We will develop an SMS scheme containing customized content for each group based on the qualitative and quantitative data outcomes.

### Clinical trials

We will conduct three clinical trials in each target groups to investigate whether the developed DATs improve adherence to treatment and virological outcomes. The study procedures can be found in the Appendix. We will conduct a non-blinded two-armed trial comparing DAT to standard care in each subgroup. Participants will be individually randomized to one of the arms in a 1:1 ratio and be exposed to the DAT for the study duration. After follow-up, we will compare the two arms’ retention in care for breastfeeding women and treatment adherence for children and adolescents.

### Ethical approval

The formative study and the trial have been separately approved by the College Research and Ethical Review Committee (CRERC) of Kilimanjaro Christian Medical University College (KCMUCo) and the National Health Research Ethics Sub-Committee (NatHREC) of the National Medical Research Institute (NIMR) of Tanzania. Furthermore, the regional representatives of the Ministry of Health and the regional medical officer have been permitted to conduct the study. Approval for the trials was requested and obtained after we had designed the customized DAT.

### Participants

We will have three different study populations, which are the following:

1. Breastfeeding mothers living with HIV with their uninfected infants (for the survey of the formative phase, we will also include pregnant women to understand their views)
2. Children aged 0–14 and their caregivers/parents
3. Adolescents aged 15–19

The inclusion criteria are as follows:

1. Mother (of HIV-exposed infant)/child/adolescent being HIV positive, with the infant of the mother not being positive
2. Attending Care and Treatment Centres (CTC) or postnatal clinics in the Kilimanjaro region at the start of the study
3. Age of mother between 18–50 years, age of children living with HIV 0–14 years and adolescents living with HIV between 15–19 years
4. Mother, caregiver/parents and adolescents willing to use RTMM and/or receive SMS
5. Mother, caregiver/parent and adolescents able to read and understand SMS
6. Mother, caregiver/parent and adolescents aged 18–19 able to understand and willing to sign the informed consent
7. Adolescents aged 15–17 able and willing to sign informed assent

The exclusion criteria are as follows:

1. Admission to a hospital at study entry
2. Participation in other trials related to adherence and retention in care
3. Previous participation in digital health research

### Intervention

The basis for the intervention is the Wisepill® device and our previously developed SMS scheme [21]. Customized SMS will be sent daily to remind taking medication. The Wisepill® device (Wisepill RT2000®), an internet-enabled medication dispenser, enables real-time and remote medication monitoring. The device consists of a box the size of a compact camera, which holds up to 30 large pills or 60 small pills in a two-partitioned container. It is manufactured by the Wisepill® company in Cape Town, South Africa. It has features to suit research and clinical trials on adherence [33, 43]. Participants will always receive a first reminder SMS half an hour before the agreed usual time of intake. A second reminder will be sent 1 h after the agreed usual time of intake in case there is no signal of the box being opened. The intervention will only be ceased upon the participant’s request or in case the investigator sees that the participant is incapable of using the intervention.

### Attending the nurse; feedback on adherence data

Nurse counsellors will recruit participants at their respective care and treatment clinics, follow standard care according to their normal schedule of care. After signing the informed consent, we will provide parents/caregivers of children and adolescents with a Wisepill® box which they will use for 12 months, while mothers will use it for 18 months.

Study nurses will attend extensive training on all study procedures. Participants will receive feedback on their adherence to ART during their clinic visit. Study nurses will share and discuss adherence reports generated by the device with the participant during the clinic visit. We will use the ‘stages of change’ model by Prochaska and Velicer [44, 45] to give tailored feedback on these reports. Feedback will be divided into phases. In the first part of the feedback session, awareness will be generated by showing the adherence report from the DAT. Study nurses will provide basic education on the importance of adherence and the potential risks of poor adherence. Participants will be allowed to share any issues they experienced during this period of using the DAT. After this phase, the nurse and participant will discuss the possible pros and cons of changing behaviour and barriers to improving adherence. Nurses may offer possible solutions, such as daily life cues that can be coupled with medication intake. The third phase of a feedback session consists of the participant setting goals for improving adherence with the nurse (goal setting). For the formative phase, a feedback session will be done once at the end of the month using the DAT. More extensive tailored feedback sessions will be done in the trial phase of the study. The information about sharing of Wisepill® data will be included in the informed consent. In addition, we will request a consent for review of participants’ medical records and for the collection of blood samples to assess viral load and adherence.

### Blinding

Due to the nature of the intervention, which is behavioural based, and the fact that assessing the outcome, which is adherence, is part of the behavioural intervention, it is impossible to blind any person in the study. During the handling of missing data, the transformation of variables, doing subgroup analyses and covariate selection, our data analysts will be blinded.

### Sample size calculation and randomization

We calculated the sample size for each group separately as the outcomes differed. In a previous study, the proportion of breastfeeding women that stayed in care was 41% in Kilimanjaro [7]*.* We want to show an improvement in the retention of 20%. We will use 1:1 randomization in the two arms. We will use 1:1 randomization in the two arms. Using the two-sided test for proportions with *α* = 0.05 and power = 80%, we will need 102 women in each arm.

The proportion of children reaching 95% adherence was 72% in a study among orphans in Dar es Salaam [2]. We want to show an improvement in this proportion of 20%. We will use 1:1 randomization in the two arms. Using the one-sided test for proportions with *α* = 0.05 and power = 80%, we will need 52 children in each arm. Considering a drop-out of 20%, we will need 65 children with their caregivers in each arm.

The proportion of adolescents reaching 95% was 67% in a study among adolescents in the Kilimanjaro region [3]. We want to show an improvement in this proportion of 20%. We will use 1:1 randomization in the two arms. Using the two-sided test for proportions with *α* = 0.05 and power = 80%, we will need 76 adolescents in each arm. Considering a drop-out of 20%, we will need 91 adolescents in each arm.

Randomization will be done using stratified block randomization on the strata of inclusion site and sex. We will use computer-generated random numbers, which were then programmed in the randomization module of the redcap. Those who enrol participants or assign the interventions only press a button in the database after entering the inclusion site and sex of the screened participant to give them the randomisation outcome and, therefore, cannot predict the allocation.

### Study outcomes

The primary study outcome for breastfeeding women is the percentage retained in care at 18-month post-partum. For children and adolescents, the primary outcome is mean adherence to ARV treatment.

Adverse events will be monitored through an adverse event form as part of the case report form. Adverse events will be recorded by the data collectors, which are mainly the nurse counsellors in the field sites. Due to the nature of the intervention, we do not expect serious events. An incidental findings policy is in place.

### Data management and analyses

We will develop a case report form (CRF) that contains information needed for study visits. During the screening, we will collect data on the date of informed consent, demographics, disease and treatment, including current antiretroviral regimen and dosing schemes. Furthermore, the phone number of the participants and treatment supporters will be recorded. In follow-up visits, we will collect data about a change in the ARV regimen. In addition, we will collect information about pharmacy refills and self-reported adherence. Viral load will be measured and recorded at study entry and after follow-up. In addition, the infant of breastfeeding mothers will be tested for HIV according to standard care at week 6, month 9 and month 18. We will use Redcap®, an open-source database management system for randomization and data management. Data will be stored anonymously; whereby personal identifiers are stored on the participant screening log. The study’s data manager oversees all procedures considering data management and randomization. We will use intention-to-treat analyses comparing the two arms on the mentioned outcomes in each sub-study. We will also use intention-to-treat analyses comparing the two arms on the mentioned primary study outcomes. Therefore, we will use chi-square analyses to compare the percentage of women retained in care at 18 months and the percentage of children and adolescents having 95% adherence during the study period of 12 months. Also, we will do Student’s *t*-tests to compare continuous variables of adherence between arms. We will compare the percentage of infants found to be HIV positive between arms by using chi-square tests.

### Roles and responsibilities

A steering committee has been set up consisting of the principal investigator (MS), co-principal investigator (KN), study doctor (IS), pharmacist (LM) and ICT person (RM). They meet on a bi-weekly basis to provide oversight of the trial progress. Furthermore, an ethical mentor is assigned to the study who will be consulted by the steering committee in case of ethical considerations. The study doctor serves as the study coordinator with the help from the pharmacist and the ICT person. In the field sites, regular nurse counsellors and physicians in caring for and treating people living with HIV are responsible for identifying potential participants, informed consent procedures and data collection. Besides the steering committee, there is a study team consisting of the steering committee, data manager, ethics mentor, research assistants, nurse and financial administrator. Each field site has been assigned a study team member responsible for overseeing the data collection processes and study procedures. Close communication with community advisory boards that exist in our field sites.

### Monitoring

An internal monitor has been assigned to assess the study data and ensure that study procedures follow protocol and good clinical practice. As we are not investigating an invasive intervention and based on recommendations from the ethical boards, we decided not to have an independent data safety monitoring board and not to perform an interim analysis. This is also because there are no anticipated problems detrimental to the participant, but please include in the protocol. However, we will use an external monitor on an ad hoc basis provided by the reciprocal monitoring scheme of the East African Consortium of Clinical Research (EACCR). There are no further procedures and no auditing trial conduct.

### Protocol amendments and deviations

In case of needed amendments to the protocol, the funder will first be asked for permission. Based on their decision, we will seek amendment permission from the local and national ethical boards. After their permission, we will implement the amended protocol using a new version number with an adapted date and add it to the trial master file and investigator site files. We will also use a protocol breach form to record any protocol deviations. In addition, mitigation plans, including refresher trainings will be implemented to reduce and avoid frequently recorded deviations.

## Discussion

### Introduction

We believe our study will demonstrate the effect of a customized DAT on adherence to treatment among breastfeeding women, children and adolescents living with HIV in Kilimanjaro, Tanzania. Furthermore, it will give more insights into the acceptability of the tools in specific groups. These results will inform policymakers to advocate for using DAT to improve adherence to treatment.

### Limitations

Opening the Wisepill® box does not necessarily imply ingestion of pills. Furthermore, the pillbox can only contain a limited number of pills. That would mean a participant will have to refill it every 2 weeks. Although we can show a possible effect, there are several groups for which the effect will be difficult to extrapolate. One group is those who are not able to read and understand SMS. Later, we may develop an interactive voice response calling system to overcome this problem. In addition, those without a phone will not be able to participate. This will be mitigated by providing them with a simple mobile phone feature. Another limitation is that in the currently proposed study, we will still work with a pre-defined SMS structure whereby participants will receive the same messages over a given period that only intervenes on missed intakes. Messages could be more personalized and tailored by using a learning mechanism that will send messages completely tailored towards the adherence pattern of a participant.

### Strengths

Strengths of our study include the formative part, in which we investigate the user’s experience of the DAT, their preference for the type of SMS to be received, and the timing of such SMS. Therefore, the intervention has been developed based on input from end-users. Another strength is that we will conduct the study in three groups, considered vulnerable and often overlooked in research.

### Dissemination

We plan to disseminate our study at different levels starting with the target group through the participants and community advisory boards. We will also disseminate results to the scientific community through peer-reviewed manuscripts and abstract presentations at conference proceedings. Nationally, we will disseminate to the Ministry of Health and National Aids Control Programme and regionally through the East African Consortium on Clinical Research (EACCR). On an international level, we will inform the World Health Organization of our results. We will develop different kinds of policy briefs for all levels to communicate and disseminate our study. In addition, the data management plan will provide for the accessibility of data and sharing. Initially, meta-data will be fully accessible without the identifiable or sensitive information about participants. The full dataset will be available upon request from the principal investigator. However, a number of conditions apply, which are (1) completion of the Kilimanjaro Clinical Research Institute (KCRI) data sharing permission form, (2) a study team member be a co-author of any planned publication on the data and (3) specific objectives to justify why sharing data is important and the advantages you anticipate to bring society at large and (4) a signed and authorities-approved data transfer agreement (DTA) form as provided by the Tanzanian national ethical review committee.

### Conclusion

Our study will give clear insights into the needs and contents of a digital tool and the effect of customization of that tool on adherence to treatment among breastfeeding women, children and adolescents living with HIV.

## Trial status

Current status: data was analysed for the formative study. We have started recruitment for the trial and expect to finalize recruitment by February 2024 and follow-up by August 2024.

## Data Availability

Data will be made available according to the Tanzanian guidelines for data sharing including using a data transfer agreement form.

## Appendix

**Table 1:** SPIRIT-figure for the schedule of enrolment, interventions, and assessments for stable children and adolescents living with HIV

	**Enrolment**	**Allocation**	**Follow-up**				**Close-out**
TIMEPOINT	***0***	**0**	***M3***	***M6***	***M9***	***M12***	***M12*** ** + **
**ENROLMENT:**							
**Eligibility screen**	X						
**Patient information and explanation**	X						
**Informed consent**	X						
***In- and exclusion criteria***	X						
**Allocation**		X					
**INTERVENTIONS:**							
***DAT with SMS reminder cues and tailored feedback***			X	X	X	X	
***Control***			X	X	X	X	
**ASSESSMENTS:**							
***Enrolment interview***		X					
***Self-reported adherence and pharmacy refill counts***		X	X	X	X	X	
***Psychosocial interview***		X		X		X	
***Viral load blood sample***		X				X	
***Exit-interview, in-depth interview and focus group discussions***							X

**Table 2:** SPIRIT-figure for the schedule of enrolment, interventions, and assessments for unstable children and adolescents living with HIV

	**Enrolment**	**Allocation**	**Follow-up**						**Close-out**
TIMEPOINT	***0***	**0**	***M2***	***M4***	***M6***	***M8***	***M10***	***M12***	***M12*** ** + **
**ENROLMENT:**									
**Eligibility screen**	X								
**Patient information and explanation**	X								
**Informed consent**	X								
***In- and exclusion criteria***	X								
**Allocation**		X							
**INTERVENTIONS:**									
***DAT with SMS reminder cues and tailored feedback***			X	X	X	X	X	X	
***Control***			X	X	X	X	X	X	
**ASSESSMENTS:**									
***Enrolment interview***		X							
***Self-reported adherence and pharmacy refill counts***		X	X	X	X	X	X	X	
***Psychosocial interview***		X			X			X	
***Viral load blood sample***		X						X	
***Exit-interview, in-depth interview and focus group discussions***									X

**Table 3:** SPIRIT-figure for the schedule of enrolment, interventions, and assessments for breastfeeding women living with HIV

	**Enrolment**	**Allocation**	**Follow-up**										**Close-out**
TIMEPOINT	***0***	**0**	***M1***	***M2***	***M4***	***M6***	***M8***	***M10***	***M12***	***M14***	***M16***	***M18***	***M18*** ** + **
**ENROLMENT:**													
**Eligibility screen**	X												
**Patient information and explanation**	X												
**Informed consent**	X												
***In- and exclusion criteria***	X												
**Allocation**		X											
**INTERVENTIONS:**													
***DAT with SMS reminder cues and tailored feedback***			X	X	X	X	X	X	X	X	X	X	
***Control***			X	X	X	X	X	X	X	X	X	X	
**ASSESSMENTS:**													
***Enrolment interview***		X											
***Self-reported adherence and pharmacy refill counts***		X	X	X	X	X	X	X	X	X	X	X	
***Psychosocial interview***		X				X			X			X	
***Viral load blood sample***		X							X			X	
***Infant HIV testing***		X					X					X	
***Exit-interview, in-depth interview and focus group discussions***													X
